# Racial Disparities in Survival Outcomes of Patients With Serous Epithelial Ovarian Cancer: A Retrospective Cohort Analysis

**DOI:** 10.7759/cureus.34389

**Published:** 2023-01-30

**Authors:** Kishan Shingala, Sarah Stavros, Sonam Parag, Abigail Tercek, Sarah S Makhani, Antoun Bouz, Alexandra Galbo, Katherine Chung-Bridges

**Affiliations:** 1 College of Medicine, Nova Southeastern University Dr. Kiran C. Patel College of Allopathic Medicine, Davie, USA; 2 Medical and Population Health Sciences Research, Florida International University (FIU) Herbert Wertheim College of Medicine, Miami, USA; 3 Research, Health Choice Network, Miami, USA

**Keywords:** serous ovarian carcinoma, overall survival (os), survival analyses, racial inequalities, high-grade serous ovarian carcinoma

## Abstract

Objective: To identify racial disparities in five-year survival rates in women affected by serous epithelial ovarian carcinoma in the United States (US).

Methods: This retrospective cohort study analyzed data from the 2010 to 2016 Surveillance, Epidemiology, and End Results (SEER) program database. Women with a primary malignancy of serous epithelial ovarian carcinoma, using International Classification of Diseases for Oncology (ICD-O) Topography Coding and ICD-O-3 Histology Coding, were included in this study. Race and ethnicity were combined into the following groups: Non-Hispanic White (NHW), Non-Hispanic Black (NHB), Non-Hispanic Asian/Pacific Islander (NHAPI), Non-Hispanic Other (NHO), and Hispanics. Cancer-specific survival was measured at five years post-diagnosis. A comparison of baseline characteristics was assessed using Chi-squared tests. Unadjusted and adjusted Cox regression models were used to calculate hazard ratios (HR) and corresponding 95% confidence intervals (CI).

Results: From 2010 to 2016, there were 9,630 women with a primary diagnosis of serous ovarian carcinoma identified in the SEER database. A higher proportion of Asian/PI women (90.7%) were diagnosed with high-grade malignancy (poorly differentiated/undifferentiated) compared to NHW women (85.4%). NHB women (9.7%) were less likely to undergo surgery when compared to NHW women (6.7%). Hispanic women had the highest proportion of uninsured women (5.9%), while NHW and NHAPI had the lowest (2.2% each). A higher proportion of NHB (74.2%) and Asian/PI (71.3%) women presented with the distant disease compared to NHW women (70.2%). After adjustment for age, insurance, marital status, stage, metastases, and surgical resection, NHB women had the highest hazard of death within five years compared to NHW women (adjusted (adj) HR 1.22, 95% CI 1.09-1.36, p<0.001). Hispanic women also had lower five-year survival probabilities compared to NHW women (adj HR 1.21, 95% CI 1.12-1.30, p<0.001). Patients undergoing surgery had significantly increased survival probability compared to those who did not (p<0.001). As expected, women with Grade III and Grade IV disease both had significantly lower five-year survival probabilities compared to Grade I (p<0.001).

Conclusion: This study reveals that there is an association between race and overall survival in patients with serous ovarian carcinoma, with NHB and Hispanic women having the highest hazards of death compared to NHW women. This adds to the existing body of literature as survival outcomes in Hispanic patients relative to NHW patients are not well documented. Because of the potential interplay between overall survival and several factors including race, future studies should aim to investigate other socioeconomic factors that may be impacting survival.

## Introduction

In 2021, the incidence and mortality of ovarian cancer in the United States (US) were 21,410 and 13,770, respectively [1]. After optimal debulking and chemotherapy, approximately 70% of patients will relapse within three years [2]. Serous epithelial ovarian carcinoma is the most common subtype of ovarian cancer and accounts for up to 90% of ovarian cancer cases [3]. Despite the steady increase in five-year survival rates of ovarian cancer in the last 30 years, survival outcomes have considerably more variability when stratified by race. The national data show that both incidence and mortality rates of patients with ovarian cancer are higher among White women compared to Black women [4]. However, multiple studies have shown that survival rates are paradoxically lower in Black women [1,5,6]. While some studies have investigated how race may impact treatment and outcomes in patients with serous ovarian carcinoma, other factors, such as income and insurance status, may also play a role.

Identifying specific disparities related to the social determinants of health is crucial in aiding clinicians in their approach to patient care. Although the existing literature has established the presence of racial disparities between Black and Non-Hispanic White women in serous ovarian carcinoma diagnosis, treatment, and survival, the data is limited regarding the differences in outcomes among other racial/ethnic minorities in the US, specifically Hispanic patients. The objective of this study was to evaluate the association between race/ethnicity and one- and five-year survival rates in women with serous ovarian carcinoma in the US during 2010-2016. This study was presented as a poster at the Society of Gynecologic Oncology Annual Meeting in March 2021.

## Materials and methods

Study design

This was a retrospective cohort study using secondary data analysis of information from the Surveillance, Epidemiology, and End Results (SEER) Program during 2010-2016. The database collects and publishes data on cancer cases from various locations and sources throughout the US to provide statistics on cancer incidence and survival data in an effort to reduce the cancer burden among the US population. Data on mortality was provided by the National Center for Healthcare Statistics and data regarding the population was obtained from the Census Bureau.

Study population

This study included adults aged 18 and older who are represented in the SEER database with a primary diagnosis of serous epithelial ovarian carcinoma using the International Classification of Diseases for Oncology (ICD-O) Topography Coding and ICD-O-3 Histology Coding from 2010 to 2016. Patients who were diagnosed at autopsy, via death certificate, or had missing data on race/ethnicity and survival were excluded from the analysis.

Variables

The primary exposure in this study was race/ethnicity. Race and ethnicity were combined into the following groups: Non-Hispanic White (NHW), Non-Hispanic Black (NHB), Non-Hispanic Asian/Pacific Islander (NHAPI), Non-Hispanic other (NHO), and Hispanic. The outcome variable was overall survival at one and five years following diagnosis of serous ovarian carcinoma. Demographic information included age at diagnosis (18 and older), insurance status (private, Medicaid, uninsured, and insurance NOS), marital status categorized as married or not married, and management with surgery defined as a simple "yes or no." The variables single (never married), separated, divorced, widowed, and unmarried partner were combined as unmarried. Tumor grade was also included using the following classifications: well-differentiated, moderately differentiated, poorly differentiated, and undifferentiated. The summary stage was incorporated to understand the extent of cancer and was classified as distant, localized, and regional. Both tumor grade and summary stage were defined according to the existing classifications within the SEER database. Finally, specifics regarding metastasis were included using the most common sites of metastasis, categorized as metastasis to bone, liver, or lung.

Statistical analyses

Data analysis was conducted using Stat/MP version 15.2 (StataCorp, College Station, TX). Baseline characteristics were reported for demographic and socioeconomic variables, reporting percentages for nominal and categorical variables. Following the descriptive statistics, a bivariate chi-squared analysis was conducted to identify possible confounders. Log-rank and Kaplan Meier curves were used to compare survival between the five racial/ethnic groups. Unadjusted and adjusted Cox regression models were used to calculate hazard ratios (HR) and the corresponding 95% confidence intervals (CI), using a p-value of 0.05 as the threshold to determine statistical significance. The proportional hazard assumptions were checked graphically.

## Results

From 2010 to 2016, a total of 9,630 subjects with a primary malignancy of serous ovarian carcinoma were identified for this study. Table 1 presents the baseline characteristics of these patients stratified by race and ethnicity. Hispanic women had the highest proportion of subjects with a primary diagnosis under the age of 50 (34.5%) while NHW subjects had the highest proportion of diagnoses over the age of 70 (28.7%). NHW subjects had the highest percentage of patients with private insurance (77.5%) and the lowest with Medicaid (7.1%). Hispanic women had the highest proportion of uninsured individuals (5.9%) while NHW and NHAPI had the lowest (2.2% each). NHAPI subjects had the highest proportion of individuals with poorly differentiated or undifferentiated cancer (90.7%) and the lowest proportion with the localized disease only (6.8%). NHB subjects had the highest proportion of individuals with distant disease (74.2%), while also having the highest proportion of individuals who did not receive surgery (9.7%).

**Table 1:** Baseline characteristics of patients with serous epithelial ovarian carcinoma by race PI: Pacific Islander, NOS: not otherwise specified

		Race/Ethnicity										
Characteristics		Non-Hispanic White		Non-Hispanic Black		Non-Hispanic Asian/PI		Non-Hispanic Other		Hispanic		p-value
		No.	%	No.	%	No.	%	No.	%	No.	%	
		6,678	69.4	694	7.2	789	8.2	114	1.2	1,355	14.1	
Survival (years)		2.4		1.9		2.1		2.2		2.1		<0.001
Age (years)												<0.001
18-49		944	15.3	147	22.8	216	28.9	25	23.2	441	34.5	
50-59		1,522	24.6	176	27.2	199	26.6	34	31.5	320	25.0	
60-69		1,947	31.5	192	29.7	190	25.4	28	25.9	309	24.1	
70-79		1,288	20.8	102	15.8	111	14.9	16	14.8	155	12.1	
80+		490	7.9	29	4.5	31	4.2	5	4.6	55	4.3	
Insurance												<0.001
Private		5,099	77.5	424	63.1	560	71.9	49	48.0	758	56.9	
Medicaid		464	7.1	130	19.4	121	15.5	31	30.4	360	27.0	
Uninsured		142	2.2	32	4.8	17	2.2	3	2.9	79	5.9	
Insurance NOS		874	13.3	86	12.8	81	10.4	19	18.6	135	10.1	
Marital Status												<0.001
Married		3,802	59.7	239	36.3	511	67.1	42	46.2	688	52.8	
Not Married		2,565	40.3	420	63.7	251	32.9	49	53.9	615	47.2	
Grade												<0.001
Well-differentiated		231	4.4	30	5.9	18	2.9	8	10.0	70	6.5	
Moderately differentiated		540	10.2	59	11.6	40	6.4	8	10.0	119	11.0	
Poorly differentiated		2,118	40.0	206	40.5	278	44.3	42	52.5	492	45.6	
Undifferentiated		2,404	45.4	214	42.0	291	46.4	22	27.5	397	36.8	
Summary Stage												0.034
Distant		4,649	70.2	510	74.2	553	71.3	68	65.4	906	67.8	
Localized		487	7.4	54	7.9	53	6.8	12	11.5	121	9.1	
Regional		1,487	22.5	123	17.9	170	21.9	24	23.1	309	23.1	
Surgery												0.053
Yes		6,228	93.3	627	90.4	739	93.7	103	92.8	1,266	93.5	
No		448	6.7	67	9.7	50	6.3	8	7.2	88	6.5	
Mets to Bone												0.313
Yes		19	0.3	5	0.7	4	0.5	0	0.0	4	0.3	
No		6,559	99.7	682	99.3	10,674	99.5	104	100.0	1,325	99.7	
Mets to Liver												0.869
Yes		340	5.2	36	5.3	45	5.9	4	3.9	72	5.4	
No		6,241	94.8	649	94.7	716	94.1	100	96.2	1,256	94.6	
Mets to Lung												0.269
Yes		234	3.6	34	5.0	35	4.6	3	2.9	49	3.7	
No		6,342	96.4	651	95.0	728	95.4	102	97.1	1,280	96.3	

**Figure 1:**
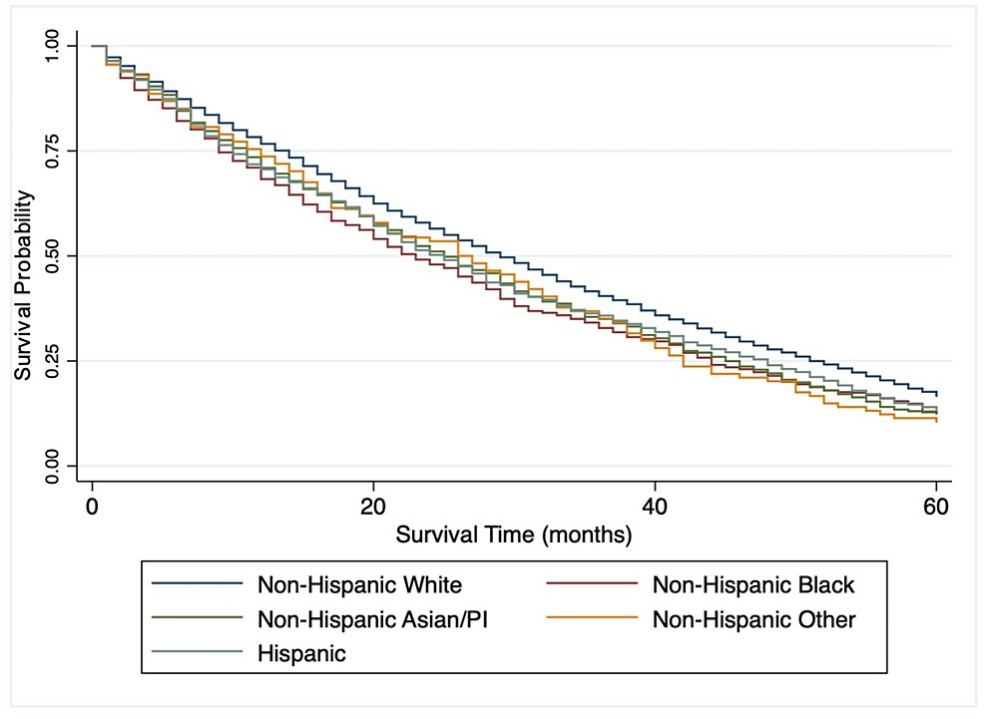
Kaplan-Meier five-year survival estimates PI: Pacific Islander

Unadjusted and adjusted Cox regression with 95% CI for one- and five-year survival using univariate and multivariate models are presented in Table 2. After adjusting for age, insurance status, marital status, tumor grade, tumor stage, surgery status, and presence of metastases, NHB subjects had a higher hazard of mortality for five-year survival compared to NHW subjects (p<0.001). Furthermore, Hispanic subjects (adj HR: 1.21, 95% CI: 1.12-1.30) and NHAPI subjects (adj HR: 1.18, 95% CI: 1.07-1.30) also had higher mortality rates than NHW subjects. A Kaplan Meier curve is displayed in Figure 1 to graphically depict the five-year survival between the five racial/ethnic groups. Those with Medicaid had a 19% increased hazard of mortality than those with private insurance (95% CI: 1.09-1.30). After adjustment, those who received surgery had an increased likelihood of survival at one and five years than those who did not (p<0.001). As expected, survival outcomes were worse for individuals over the age of 70 years (p<0.001), those with Grade III or Grade IV cancers (p<0.001), and those with distant spread of disease (p=0.002) compared to their counterparts.

**Table 2:** Unadjusted and adjusted Cox regression with 95% confidence intervals for one- and five-year survival *Adjusted for age, insurance, marital status, insurance status, grade, stage, and surgical intervention, CI: confidence interval, HR: hazard ratio, Ref: reference, PI: Pacific Islander, NOS: not otherwise specified

	One-Year Survival					Five-Year Survival				
Characteristics	Unadjusted		Adjusted*			Unadjusted		Adjusted		
	HR	(95% CI)	HR	(95% CI)	p-value	HR	(95% CI)	HR	(95% CI)	p-value
Race										
Non-Hispanic White	Ref		Ref			Ref		Ref		
Non-Hispanic Black	1.43	(1.24-1.65)	1.39	(1.15-1.68)	0.001	1.22	(1.12-1.32)	1.22	(1.10-1.36)	<0.001
Non-Hispanic Asian/PI	1.28	(1.11-1.47)	1.30	(1.10-1.54)	0.003	1.17	(1.08-1.28)	1.18	(1.08-1.30)	0.001
Non-Hispanic Other	1.16	(0.81-1.66)	0.99	(0.59-1.69)	0.995	1.22	(1.01-1.49)	1.25	(0.97-1.62)	0.090
Hispanic	1.30	(1.17-1.45)	1.46	(1.27-1.67)	<0.001	1.15	(1.08-1.22)	1.21	(1.12-1.30)	<0.001
Age (years)										
<50	Ref		Ref			Ref		Ref		
50-59	1.01	(0.94-1.22)	1.15	(1.15-1.68)	0.078	1.03	(0.97-1.11)	1.04	(0.96-1.13)	0.306
60-69	1.19	(1.04-1.34)	1.22	(1.09-1.54)	0.011	1.15	(1.07-1.23)	1.12	(1.03-1.21)	0.006
70-79	1.44	(1.26-1.64)	1.45	(0.59-1.69)	<0.001	1.29	(1.20-1.39)	1.20	(1.10-1.32)	<0.001
80+	2.08	(1.77-2.45)	1.71	(1.27-1.67)	<0.001	1.64	(1.48-1.81)	1.40	(1.24-1.59)	<0.001
Insurance										
Private Insurance	Ref		Ref			Ref		Ref		
Medicaid	1.34	(1.19-1.51)	1.28	(1.11-1.50)	0.001	1.22	(1.14-1.30)	1.21	(1.11-1.31)	<0.001
Uninsured	1.14	(0.90-1.43)	1.00	(0.73-1.36)	0.994	0.89	(0.78-1.02)	0.89	(0.74-1.02)	0.093
Insurance NOS	0.98	(0.86-1.11)	0.92	(0.79-1.08)	0.300	1.03	(0.96-1.10)	1.02	(0.94-1.11)	0.648
Marital Status										
Married	Ref		Ref			Ref		Ref		
Not Married	1.19	(1.09-1.29)	1.03	(0.93-1.14)	0.570	1.13	(1.07-1.18)	1.06	(1.00-1.12)	0.036
Grades										
Grade I	Ref		Ref			Ref		Ref		
Grade II	1.06	(0.79-1.43)	1.17	(0.85-1.61)	0.333	1.08	(0.94-1.25)	1.08	(0.92-1.26)	0.343
Grade III	1.27	(0.98-1.64)	1.35	(1.01-1.79)	0.040	1.28	(1.13-1.45)	1.22	(1.07-1.40)	0.003
Grade IV	1.64	(1.27-2.11)	1.81	(1.37-2.40)	<0.001	1.55	(1.37-1.76)	1.52	(1.33-1.74)	<0.001
Stage										
Distant	Ref		Ref			Ref		Ref		
Localized	1.02	(0.88-1.18)	1.38	(1.14-1.66)	0.001	0.77	(0.71-0.85)	0.90	(0.81-1.00)	0.053
Regional	0.96	(0.87-1.06))	1.12	(0.99-1.26)	0.064	0.82	(0.78-0.86)	0.89	(0.83-0.95)	<0.001
Surgery										
No	Ref		Ref			Ref		Ref		
Yes	0.34	(0.31-0.39)	0.34	(0.28-0.43)	<0.001	0.47	(0.43-0.51)	0.52	(0.44-0.61)	<0.001

## Discussion

This study demonstrated an association between race/ethnicity and cancer-specific survival outcomes for patients with a primary diagnosis of serous epithelial ovarian carcinoma. Hispanic, NHB, and NHAPI respondents appear to have a higher risk of mortality over both a one- and five-year follow-up period after diagnosis compared to NHW individuals. After adjusting for age, insurance status, marital status, tumor grade, tumor stage, surgery status, and presence of metastases, Hispanic women had the lowest survival at one year and NHB women had the lowest survival at five years. Women of minority ethnic and racial backgrounds may face significant barriers to receiving a timely diagnosis and appropriate treatment for serous ovarian carcinoma, both of which are critical for improving patient survival. The findings in the present study are somewhat consistent with the existing literature. Several studies have shown that Black women have higher serous ovarian carcinoma-related mortality than White women [6-10]. However, it has been reported in other research that survival is better in Hispanic and NHAPI women than in NHB and NHW women [11]. Additionally, one study reports that after adjusting for stage, optimal surgical debulking, and platinum sensitivity, there was no difference in survival between NHB and NHW women [12].

The underlying causes of the observed disparities are equivocal and likely multifactorial. Literature has also shown that Black women are more likely than White women to be diagnosed at younger ages, with undifferentiated and unclassified cancers, and with distant and metastatic disease [8,13-15]. One retrospective cohort analysis found that differences in survival between NHB and NHW patients exist with the regional disease but not the distant disease, suggesting that extent of the disease acts as an effect modifier [16].

It has been shown that Black patients with serous ovarian carcinoma are less likely to receive guideline-based treatment than White patients including surgical resection of primary disease, lymphadenectomy in conjunction with their primary surgery, and surgery-chemotherapy sequences [5,6,8,10]. One study found that there was an independent increase in the risk of non-guideline concordant care of 53% for NHB and 31% for Hispanic patients when compared to NHW patients and that guideline-concordant care is associated with improvement in survival of ovarian cancer [13]. Another study found that after adjusting for guideline-concordant care, differences in survival between races disappeared, suggesting access to care is partially responsible for racial differences in survival [12].

Observed disparities may also be mediated by insurance status, which is often linked to socioeconomic status. Our study found that patients with Medicaid had a 19% increased hazard of mortality compared to those with private insurance (95% CI: 1.09-1.30), and baseline characteristics reveal that larger proportions of NHB, Hispanic, and NHAPI patients utilized Medicaid. A study analyzing prognostic factors in the survival of ovarian cancer found that long-term survivors are more likely to be insured [17]. These findings could help explain why our study found decreased survival for NHB and Hispanic women in comparison to NHW women, as well as why these populations are more likely to present with more advanced diseases. While the aforementioned study found that NHB women had a higher risk of mortality than NHW women for the first six years following diagnosis, the association declined over time and there was no difference in survival between nine and 13 years following diagnosis [17]. This could potentially be due to access to care and receipt of guideline-concordant therapy, which may differentially impact survival in the short term rather than the long term. However, it must also be considered that women that survive longer have other cancer-related factors that provide a better prognosis or that differences in survival may not be significant because the sample size has become too small to detect a difference. Additionally, a retrospective cohort using data from the SEER database also found lower rates of insurance in NHB than in NHW patients but found no significant effect of insurance status as a modifier of survival [16].

In addition to socioeconomic factors, there likely is an interplay between biologic and non-biologic factors that contributes to racial disparities in the survival of ovarian cancer. Current literature suggests that genetic mutations and polymorphisms, as well as epigenetic changes such as differential methylation and alteration in gene expression, have a role in the outcomes of ovarian cancer [18]. One example of this is the differential overexpression of phosphoserine phosphatase homolog (PSPHL) in the ovarian cancers of NHB patients in comparison to NHW patients [19].

Limitations were introduced by the nature of the study design; retrospective cohort studies limit the variables that are accessible for use, potentially leaving out key statistics that may be pertinent to the study’s conclusions. The SEER database does not include data regarding patient income, which serves as an indicator of socioeconomic status. However, the analysis did include data on insurance coverage, which is a surrogate marker for socioeconomic status. The analysis also lacks data about patient comorbidities, which affect surgical candidacy, recurrence, and, ultimately, survival. We also did not have data regarding access to care, patient preferences, and the recommended and received treatments. The external validity is also limited as the SEER database only includes 34% of all cancers. Thus, the characteristics of the women included in our study may not be representative of the racial and ethnic populations nationally. It is important to incorporate more representation in future studies to optimize external validity.

## Conclusions

The findings from this study, in conjunction with the current literature, suggest that racial disparities impact the overall survival in patients with serous ovarian cancer, with NHB and Hispanic women having lower one- and five-year survival rates compared to NHW women. Documentation of differences in survival by race/ethnicity is critical, as it demonstrates a need for further investigation into the socioeconomic factors contributing to this disparity so that they might be addressed.
